# Microstructural Assessment of Pozzolanic Activity of Ilmenite Mud Waste Compared to Fly Ash in Cement Composites

**DOI:** 10.3390/ma17112483

**Published:** 2024-05-21

**Authors:** Filip Chyliński

**Affiliations:** Instytut Techniki Budowlanej, Filtrowa 1, 00-611 Warsaw, Poland; f.chylinski@itb.pl

**Keywords:** microstructure, titanium dioxide waste, ilmenite mud waste, cement composites, fly ash, pozzolanic activity, thermal analysis, R^3^ test

## Abstract

This paper presents the influence of adding rinsed ilmenite mud waste (R-MUD) on the microstructure of Portland cement composites, compared to similar composites containing fly ash (FA). The aim of the study is the assessment of the pozzolanic activity of ilmenite mud waste by its impact on the microstructure of the cement matrix in comparison to the undoubted pozzolanic activity of fly ash. The presented test results include pore size distribution, phase composition, pozzolanic activity using thermal analysis, R^3^ bound water test, and microstructural analysis using scanning electron microscopy (SEM). Tests were performed on mortars cured for up to 360 days. The results presented in this paper have shown that R-MUD has a pozzolanic activity level similar to FA or better, which influences pore size distribution in the composite and its microstructure. During the curing process, the microstructure of composites containing R-MUD became more compact and sealed than those with FA, which might also increase their durability. The results of the R^3^ tests have proven the pozzolanic activity of R-MUD but its level was lower than FA. R-MUD might be a useful substitute for fly ash, especially given the lack of good-quality fly ash on the market.

## 1. Introduction

**Figure 1:**
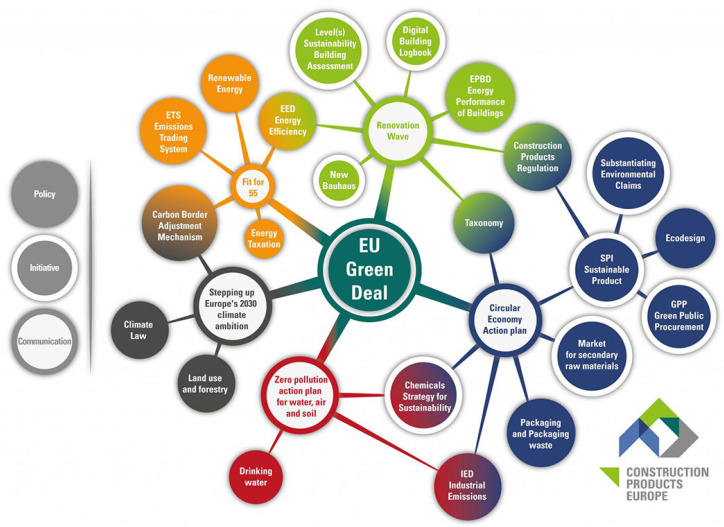
The European Green Deal [4].

Due to climate change, many countries have developed their own strategies to decrease the emission of greenhouse gases [1]. Europe has become a leader in the field of green politics by signing, in 2021, the EU Green Deal (EUGD) [2]. The aim of this strategy is to achieve climate neutrality by the year 2050. In the construction field, with its high environmental impact, many processes have to be substantially changed [1,3]. The use of resources and the harmonisation of product manufacturing and construction activities is described in the Industrial Strategy, which is part of the EUGD strategy [4]. Concrete production has one of the largest environmental impacts in the whole construction sector, mainly due to the production of the Portland cement clinker estimated for about 4.2 billion tons, and due to the high demand for concrete in the market, especially in developing countries. The EUGD strategy has affected developments in the construction sector, as it mandates increased sustainability and the use of secondary raw materials, as per the Circular Economy Action plan, which is a part of the EUGD (Figure 1).

The main issue in sustainable development is to use waste and recycled materials instead of natural resources as much as possible, all the while remembering that the functional properties of the final construction product should be similar to the reference one and that any deterioration of performance characteristics should be within acceptable limits [5,6,7]. The production of Portland cement clinker has one of the highest emission rates of carbon dioxide in the construction sector. The cement industry contributes more than 5% of the total anthropogenic emissions of CO_2_ [8,9,10,11,12,13,14,15,16]. The cement production sector is already using large amounts of various types of waste and by-products, including granulated blast furnace slag, fly ash, and silica fumes to produce different types of cement mixes with a lower carbon footprint [6,17,18,19]. The current European standards also give the green light for the production of low clinker cements, such as those described in European standard EN-197-5 [20], which allows for the production of cements with a clinker content of 50% or 35% in composite cements CEM VI containing ground granulated blast furnace slag. Another European standard being prepared is EN-197-6 [21], which will allow for the use of industrial concrete fines, recycling up to 20% of cement composition. 

The EUGD strategy strongly impacts various industrial sectors, which also affects those by-products already successfully used in the concrete industry, such as fly ash (FA). Fly ash is a by-product derived from power plants when coal dust is combusted to generate heat and electrical power. The most useful fly ash for concrete is that generated when combusting hard coal. Once fly ash fulfils the requirements of the EN 450-1 standard, it becomes a very valuable building material especially for the cement and concrete industries [22,23,24]. Not all fly ash available in the market, however, is generated from combusting hard coal. Some is produced when burning lignite, municipal waste, wood waste, and various other types of waste. The requirement for successively lowering CO_2_ emissions led to an increased demand for good-quality fly ash on the market, which has caused a deficit. This situation creates an opportunity to develop new types of useful additives in cement or concrete production, which will be able to cover the shortage of fly ash on the market [18,25,26].

A good way of fulfilling the EUGD strategy is taking waste generated in one industry and reusing it in another as secondary raw material. The waste on which this article is focused is ilmenite mud, which is generated during the production of titanium dioxide.

Titanium dioxide is a white pigment widely used in the production of paints, catalytic coatings, plastics, paper, pharmaceuticals, and sunscreen, but it might also have other applications such as in packaging, cosmetics, and food [27]. The annual production of titanium dioxide was estimated as roughly five million tons in 2021 with an approximately 5% growth rate [28]. Titanium dioxide is produced in two main ways, either by using sulphide or chloride. Each of these methods has its advantages and disadvantages but this paper focuses on waste generated using the sulphide method. Ilmenite mud waste is a residue material produced after leaching ilmenite with highly concentrated sulphuric acid. Producing titanium dioxide using the sulphide method might lead to the generation of 0.8 million tons of hazardous ilmenite mud waste, which in most cases is being placed in landfills [29]. Previous tests have shown that hazardous ilmenite mud valorised directly in a factory had the potential of becoming a safe and valuable material, namely rinsed-MUD (R-MUD). Scholars have proven that R-MUD has a pozzolanic activity of the same level or better as fly ash and can be used as an additive in cement composites, including mortars and concretes, with functional properties and durability not worse than composites containing the same amount of fly ash [30,31]. However, the results of the published tests do not explain why the additive with relatively low content of reactive silicon dioxide comparing to the FA, has similar pozzolanic activity or even better. There are no available publications about this type of waste material which might explain this phenomenon which also speaks to the novelty of this paper.

Substituting fly ash with a different type of material necessitates the investigation of not only the functional properties but also the durability of the newly prepared composites. Previous experiments have shown that it is possible to obtain a cement composite containing R-MUD which is durable in several types of aggressive environments [31,32]. There have been no studies, however, as to why this material shows relatively high pozzolanic activity or how its addition increases the durability of concrete. The content of silicon dioxide in R-MUD is much lower than in siliceous fly ash; however, R-MUD shows pozzolanic reactivity at similar level or even better than FA. Assessment of pozzolanic activity of material also depends on the type of test method used.

The aim of the study is the assessment of the pozzolanic activity of ilmenite mud waste by its impact on the microstructure of the cement matrix in comparison to the undoubted pozzolanic activity of fly ash. This paper presents a comparative assessment of pozzolanic activity and its influence on the microstructure of cement composites containing R-MUD in reference to composites with FA, using the following test methods:

- X-ray diffraction (XRD)
- differential thermal analysis (DTA)
- R^3^ bound water
- mercury intrusion porosimetry (MIP)
- scanning electron microscopy (SEM)

XRD, DTA, and MIP analysis were also performed after various times of curing (7, 14, 28, 90, 180, and 360 days) to observe the changes in the analysed properties over time. The aim of the XRD tests was to determine the phase composition of mortars and its semi-quantitative changes over time, as well as changes in the amorphous phase amount by the analysis of the background. Thermal analysis tests were performed to determine more precisely the changes in quantities of the constituents (mainly portlandite and calcite) during the time of curing. R^3^ bound water test was performed to prove the pozzolanic activity of R-MUD and compare it to the activity of FA, as well as to the results of previously performed experiments. MIP analysis was carried out to show how pore distribution in the cement matrix changes during the time of curing and compare both R-MUD and FA. SEM examinations were carried out on samples after 360 days of curing and its aim was to determine the differences between microstructure of both types of mortars, especially by analysing the C-S-H phase.

The main issue on which this article is focused is the evaluation of porosity over time for the cement matrix, and the transition zone between cement grout and aggregate grains as an effect of pozzolanic activity, which is one of the main factors behind the durability of this type of cement composites.

## 2. Materials and Methods

### 2.1. R-MUD and FA

The composition of the R-MUD, FA, and cement used for the experiments was tested previously [31] and is presented in Table 1.

XRD phase composition studies of R-MUD published previously [34] showed the presence of both titanium compounds and crystalline phases of relict gangue minerals. The titan-bearing phases included anatase, rutile, and ilmenite. Non-deposit minerals were represented by orthopyroxenes and plagioclases. The presence of zircon was confirmed, quartz was present only in trace amounts. The possible presence of iron(II) sulphate heptahydrate has not been ruled out. The silica present in the waste was not clearly recorded in the diffractograms, which proves its amorphous or even colloidal nature, which positively affects its reactivity. XRD tests showed that the magnesium contained in the sludge is mostly bound in the form of orthopyroxenes, silicates, which should be unreactive towards the cement matrix.

### 2.2. Mortars

The composition of mortars used for the tests is presented in Table 2. The base composition of the mortar was a standard mortar according to EN 196-1 [35], modified by replacing 10.8% by mass of cement with R-MUD or FA. The amount of 10.8% is the optimal value indicated by previous tests and analysis [36].

A set of mortar samples with the dimensions 40 × 40 × 160 mm were moulded. After 24 h, they were demoulded and cured in a chamber at a temperature of 20 ± 2 °C and humidity above 95%. After a period of time (7, 14, 28, 90, 180, and 360 days), two samples for each type of composition were taken out of the chamber. From each sample, three small cylinders (diameter 25 mm, height 35 mm) were drilled for porosity tests. These were then dried in an oven at 40 °C for 24 h. Next, they were placed into 99.8% ethanol in vacuum (30 mbar) for another 24 h to stop the hydration. Then, they were dried in an oven at 40 °C in vacuum (20 mbar) for 24 h. The samples for the XRD and DTA tests were ground to a particle size of less than 0.063 mm.

### 2.3. X-ray Diffraction

Powdered mortar samples were analysed using the diffractometer model D8 Advance eco produced by Bruker (Billerica, MA, USA). CuKα X-ray was used with a voltage of 40 kV and a current of 25 mA. Diffractograms were collected in a range of 2theta 6–66° with a step of 0.01° and a time of 5 s for each step, using the LYNXEYE XE-T (SSD 160-2) detector. Diffractograms were analysed using the DIFFRAC.EVA v. 6.0 software with the PDF-2 ICDD 2023 database.

### 2.4. Differential Thermal Analysis

Ground samples of mortar for each curing period were analysed using the thermal analyser model SDT Q600 produced by TA Instruments (New Castle, DE, USA). The weight of each tested sample was about 20–30 mg. The samples were analysed in a platinum crucible in temperatures ranging from 20 to 1000 °C, using a heat rate of 10°/min in an atmosphere of air and with an air flow of 100 mL/min. The TG, DTG, and DTA curves were collected.

### 2.5. R^3^ Bound Water Test

R^3^ bound water test was carried out according to the Report of RILEM TC 267-TRM [37,38]. The method of testing the activity of supplementary cementitious materials (SCM) is based on the preparation of the SCM’s paste by mixing it with calcium hydroxide, water, and other constituents. Pastes were cured in a sealed container for 7 days at a temperature of 40 °C. After that, they were ground into particles of less than 2 mm and dried until constant mass in a temperature of 40 °C and weight. The next step was heating the samples in a furnace at a temperature of 350 °C until a constant mass. As a result of the test, the water content which evaporates during the heating was determined and it is expressed as a g_H2Obound_/100 g of dried paste in 40 °C.

### 2.6. Mercury Intrusion Porosimetry

The pore distribution of mortars was analysed on cylindrical samples using the mercury porosimeter model PM608, produced by Quantachrome Instruments (Anton Pear Group, Boynton Beach, FL, USA), which allows for the analysis of pores with a diameter of larger than 3.6 nm. Each type of mortar, after each period of curing, was analysed using three samples, calculating the average values. According to the derived data, pore sizes were divided into the following groups depending on their diameter [39]:

- macropores: above 10 μm
- large capillary pores: 0.05–10 μm
- small capillary pores: 0.01–0.05 μm
- gel pores: under 0.01 μm

Also, other parameters were studied such as total porosity, pore size range, average pore volume and diameter, and median pore volume, and diameter.

### 2.7. Scanning Electron Microscopy (SEM)

Microstructure SEM examinations were undertaken on mortars with R-MUD and FA after 360 days of curing. Four mm thick slices of mortar were cut from the inner section of samples that had been cured for 360 days. These were then dried, ground, and polished in the same way as in previous experiments [32]. SE and BSD images were collected using the scanning electron microscope model Sigma 500 VP produced by Zeiss (Jena, Germany). EDX analysis and maps were drawn using the Ultim Max 40 detector.

## 3. Results and Discussion

### 3.1. X-ray Diffraction

The aim of the XRD tests was to determine the phase composition of mortars containing R-MUD and FA after different time periods of curing. The analysis focused on changes over time in different types and amounts of phase compositions. In particular, the content of the product of hydration and pozzolanic reaction was investigated, such as portlandite, calcium silicates, and aluminium silicates. Due to the fact that the C-S-H phase is mostly amorphous, it is very difficult to identify it using this method. The XRD method is used by some authors in order to determine the pozzolanic reaction; however, other studies describe this method as more qualitative than quantitative [23,40,41,42,43]. In addition, the amount of calcite was analysed to check whether the samples were equally carbonated, which might suggest that they were prepared in the same way and cured in the same conditions.

#### 3.1.1. R-MUD

**Figure 2:**
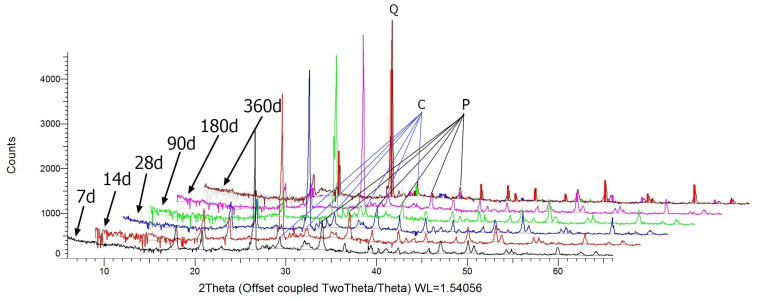
Diffractograms of mortars containing R-MUD (main peaks of Q-Quartz, P-Portlandite, and C-Calcium).

Figure 2 presents the diffractograms of mortars containing R-MUD after different periods of curing, ranging from 7 days up to 360 days.

The main crystal phases identified in the mortars containing R-MUD are quartz PDF-2 00-005-0490), portlandite (PDF-2 00-001-1079) and calcite (PDF-2 00-005-0586). Minor amounts of gypsum (PDF-2 01-072-0596), ilmenite (PDF-2 01-070-6282), and larnite (PDF-2 0-049-1673) have also been identified. It has been observed through the diffractograms that as the curing time of the samples increased, the intensity of portlandite reflexes became weaker. As the calcite reflexes are almost even in each sample, the decrease in the amount of portlandite cannot be explained by the carbonation of mortars.

The content of the amorphous phase calculated by the height of the background shows that as curing time increased, the content of the amorphous phase also increased. This might be caused by the increasing amount of the C-S-H gel phase, which is a product of the pozzolanic reaction. Also, the presence of a calcium silicate phase (larnite) has been observed, which might be a clinker relic due to its high similarity to the belite structure. The correlation between the intensity of those reflexes as curing time increases is not clear but may be caused by the relatively low intensity of reflexes from this phase.

#### 3.1.2. FA

**Figure 3:**
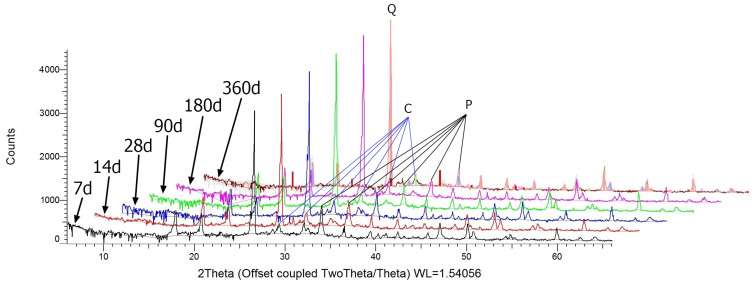
Diffractogram of mortars containing FA (main peaks of Q-Quartz, P-Portlandite, and C-Calcium).

Figure 3 presents the diffractograms of mortars containing FA after different curing periods ranging from 7 days up to 360 days.

The main crystal phases identified in the mortars containing FA are quartz (PDF-2 00-046-1045), portlandite (PDF-2 01-087-0673), calcite (PDF-2 01-080-9776), and minor amounts of mullite (PDF-2 00-001-0613) and aluminium silicate (PDF-2 00-016-0602). Closely inspecting the diffractograms of FA mortars, one can observe that as the curing time of the samples increases, the intensity of the portlandite reflexes becomes weaker. On the contrary, calcite reflexes are almost even in each sample and, thus, the decreasing amount of portlandite cannot be explained by the carbonation of those mortars.

The content of the amorphous phase calculated by the height of the background shows that as curing time increased, the content of the amorphous phase also increased. This might be caused by the increasing amount of the C-S-H and C-A-S-H gel phases, which are products of the pozzolanic reaction. Also, the presence of an aluminium silicate phase has been observed, which might be a relic of FA activity. The correlation between the intensity of those reflexes and curing time of the mortars is not clear but may be caused by the relatively low content and crystallinity of this phase.

### 3.2. Differential Thermal Analysis (DTA)

The main aim of DTA tests was to determine the changes in the portlandite content in mortars, according to curing time. Thermogravimetric analysis also allows for the comparison of the amount and type of the C-S-H gel and the content of calcite generated during the carbonisation of the mortars. Differential thermal analysis is quite often used to compare the pozzolanic activity of various types of material, with better quantitative results than the XRD method [23,30,42,44,45,46].

#### 3.2.1. R-MUD

**Figure 4:**
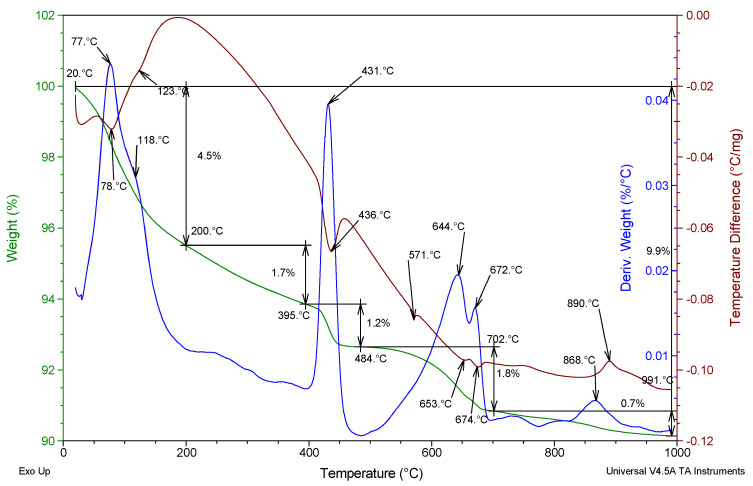
TG, DTG, and DTA thermograms of R-MUD mortar cured for 360 days (green, blue and brown curve respectively).

Figure 4 presents an example of the TG, DTG, and DTA curves of the R-MUD mortar cured for 360 days.

In the temperature range of between 20 and 200 °C, exothermic signals were registered that related to the loss of free water and water bonded in the C-S-H phase [47]. In the temperature range of between 400 and 450 °C, a high exothermic signal with a maximum at 431 °C was registered that related to the mass loss due to the decomposition of portlandite. In the temperature range of between 500 and 700 °C, loss of mass related to the decomposition of calcite was registered. Two local maxima at 644 and 672 °C might be caused by the different crystal sizes related to the different energy of activation of the decomposition reaction [23]. In the range of 800–900 °C, an endothermic effect with a maximum at 868 °C was registered, related to the decomposition of calcium silicates [48]. An exothermic signal with a maximum at 571 °C was also registered without any mass loss and is related to the crystallinity transformation of quartz.

#### 3.2.2. FA

**Figure 5:**
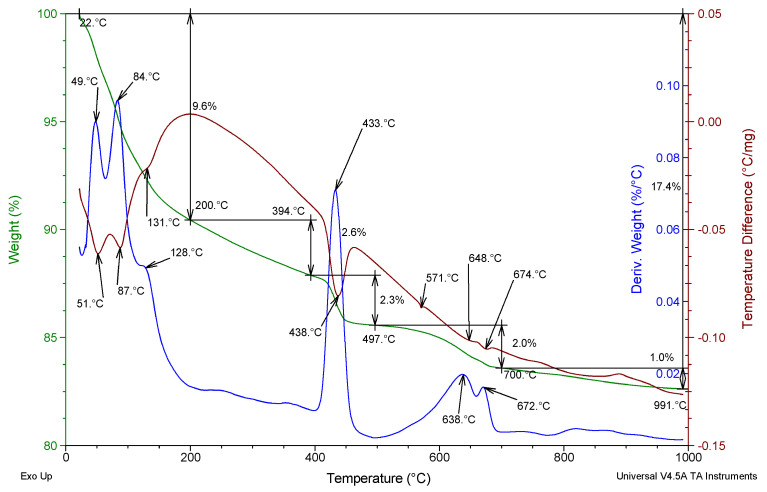
TG, DTG, and DTA thermograms of FA mortars cured for 360 days (green, blue and brown curve respectively).

Thermograms of FA mortar cured for 360 days are presented in Figure 5 and are quite similar to the R-MUD ones. The composition of the C-(A)-S-H phase is different in FA mortar than in R-MUD, with two exothermic peaks with local maxima at 49 °C and 84 °C [47]. Also, there is more free water and water bonded into the C-(A)-S-H phase, which is related to the mass loss of 9.6% in temperatures up to 200 °C. In the R-MUD sample, this was 4.5%. Differences are related to the different types of gel phase and their ability to bond with water. In R-MUD, this phase seems to be less water-permeable, while also absorbing less water. Loss of mass related to the presence of portlandite (400–500 °C) in FA mortar is higher than in R-MUD (2.3% and 1.2%, respectively), which shows that the effectiveness of the reaction in FA with portlandite is lower than in R-MUD. The level of carbonisation in both samples is about equal, as indicated by the mass loss in the range of 500–700 °C (2.0% and 1.8% for FA and R-MUD, respectively). The mass loss between 700 °C and 1000 °C is related to the decomposition of calcium silicates [48].

The data from the thermal analysis tests determined the amount of portlandite and calcite in each sample after each curing period. In order to compare those values more effectively, an equivalent of portlandite was calculated by adding a part of portlandite which had reacted to form calcium carbonate. The values obtained are presented in Figure 5.

**Figure 6:**
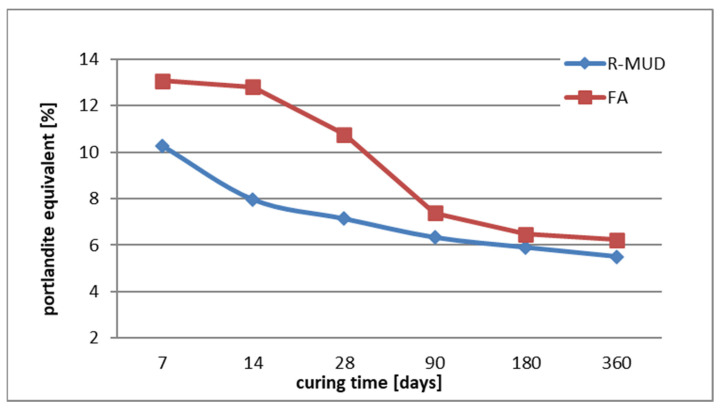
Changes in portlandite equivalent content over time.

Analysing the data presented in Figure 6, it can be seen that the amount of portlandite at 7 days in the R-MUD sample is already a few percentage points lower than in the FA, which suggests that the initial activity of the R-MUD is much higher than that of the FA [30,33]. During almost the entire curing timeframe of 360 days analysed, the amount of portlandite in both types of mortar is decreasing, which is caused by the pozzolanic reaction of the R-MUD and FA. The speed of the pozzolanic reaction in the R-MUD mortar is higher than in the FA mortar during the first 14 days. Then, the speed of the pozzolanic reaction increases in the FA mortar and slows down after day 90 all the way to day 360. At the same time, the amount of portlandite stabilises almost at a constant level between day 180 and day 360. In the R-MUD mortar sample, after 90 days, the speed of the pozzolanic reaction becomes constant and the amount of portlandite does not seem to stabilise after day 360, which might suggest that this reaction would go further. On day 360 of curing, the amount of portlandite equivalent in both mortars is almost at the same level, which shows that after a year, their pozzolanic effectiveness is on a par.

### 3.3. R^3^ Bound Water Test

Table 3 presents results of R^3^ bound water test performed for the R-MUD and FA.

**Figure 7:**
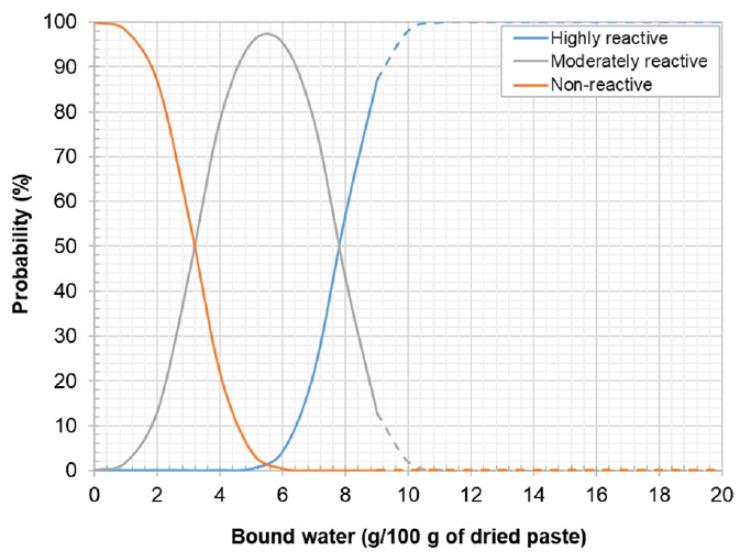
Criteria for assessing the activity of tested material [38].

Analysing the results of the R^3^ bound water tests from Table 3, it can be seen that FA has more water bound than R-MUD (7.53 and 6.12, respectively) which means that the C-S-H phase present in FA paste has an ability to bond more water what is related to its potential activity. To assess the results of the performed tests, the requirements of RILEM TC-267 for pozzolanic materials are presented on Figure 7 [38].

Comparing the results of the tests to the requirements of RILEM TC-267 presented on Figure 6 [38], it might be assessed that R-MUD is a moderately reactive material at a probability of almost 100%. However, FA has a higher reactivity, and it is assessed as a moderately reactive and highly reactive material with a probability of about 50% for each. Comparing those values with tests performed using thermogravimetric method described in Section 3.2 or results of previously performed tests [30,33] shows that FA seems to be more active material than R-MUD. Results of R^3^ bound water tests proves the pozzolanic activity of R-MUD, although differences between results of tests received using other methods needs further investigations as it might be that not all methods are accurate for testing this type of material.

### 3.4. Mercury Intrusion Porosimetry (MIP)

The aim of MIP tests was to determine the pore size distribution in mortars and study its changes over time, with specific emphasis to changes during the pozzolanic reaction process. Also, differences in the efficiency of sealing the microstructure were observed between the R-MUD and FA mortars.

Figure 7 presents changes in the total porosity of mortars depending on curing time.

**Figure 8:**
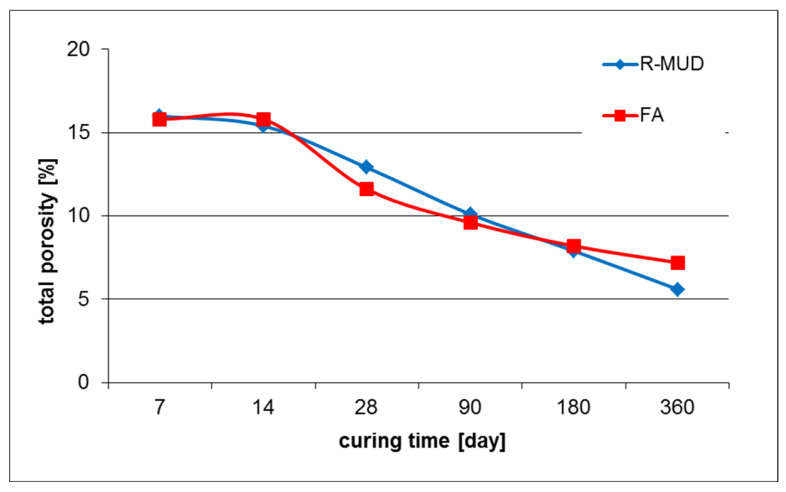
Total porosity of mortars according to different curing times.

Plots presented in Figure 8 show how the total porosity of R-MUD and FA mortars changes over time. The initial porosity of both mortars after 7 days of curing is almost equal (about 16%). Up to day 14, porosity slightly decreases in the R-MUD sample and is about constant in FA, which might suggest the initiation of pozzolanic reaction in the R-MUD sample [30,33,46]. After day 14, total porosity drops almost linearly to a value of about 5% in day 360 and does not appear to slow down, which suggests that it might drop further. Sealing of the microstructure is observed as the rate at which the total porosity decreases speeds up in the FA sample after day 14 of curing, and it seems to have the highest speed on day 28. After that, it slows down to a value of about 7% after 360 days of curing. The asymptotic shape of the FA curve suggests that the pozzolanic reaction of FA mortars will not propagate.

#### 3.4.1. R-MUD Mortars

**Figure 9:**
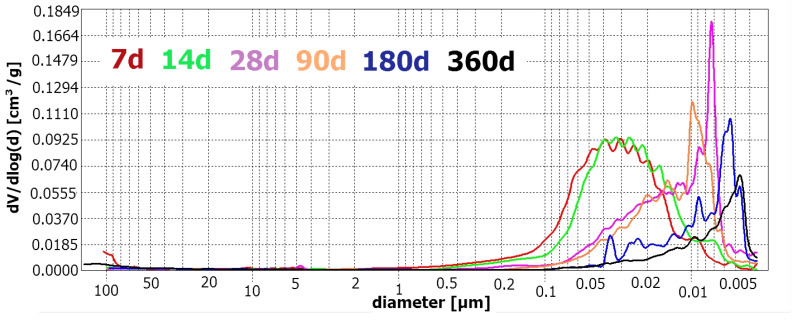
Differential pore distribution as a function of pore size diameter—R-MUD mortars.

Figure 9 presents an overlay of differential pore size distribution of R-MUD mortars cured for various lengths of time.

**Figure 10:**
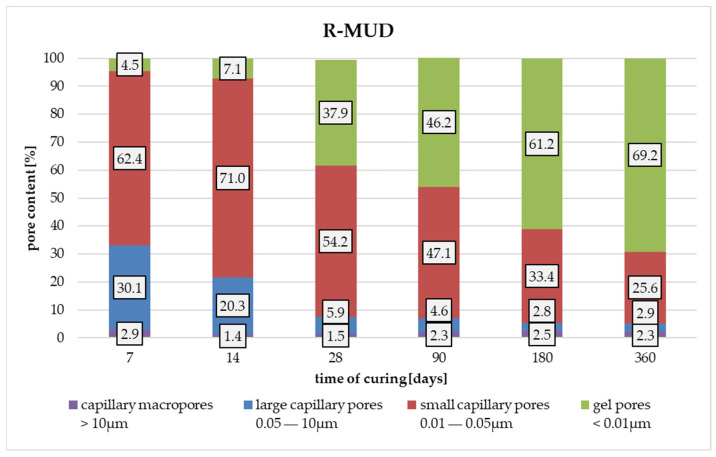
Data from the R-MUD mortar tests.

Analysing the plots in Figure 9, changes in pore size distribution depending on curing time are observed. Samples cured for up to 14 days have a relatively high number of large capillary pores in the range of 1 µm to 0.01 µm. Between days 14 and 28 of curing, pore size distribution rapidly changes, and large capillary pores are transformed into small capillary pores and gel pores, with the highest amount of pores having a diameter of about 0.07 µm. Further increasing curing time succeeded in effectively sealing the microstructure, decreasing the amount of capillary pores and increasing the amount of gel pores. Figure 10 presents the experimental results.

#### 3.4.2. FA Mortars

**Figure 11:**
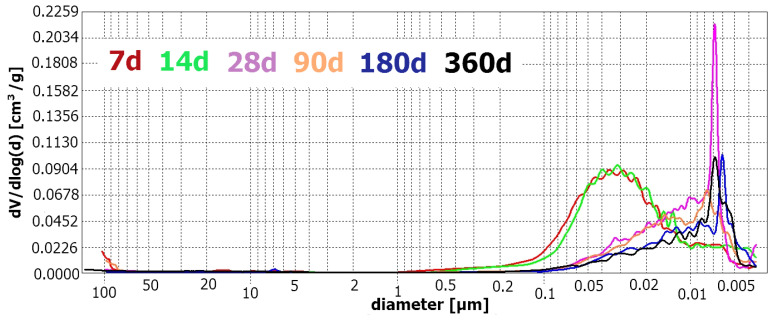
Differential pore distribution as a function of pore size diameter—FA mortars.

Figure 11 presents how the amount of each type of pore changes in total porosity depending on time of curing. It can be observed that the process of sealing the microstructure speeds up prior to day 14 of curing when the amount of gel pores increases from 7.1 to 37.9% for days 14 and 28, respectively. The influence of fly ash on the mechanical properties of mortars is observed mostly after day 28 or even day 90 of curing but, as the MIP test results have shown, the pozzolanic reaction might start earlier [23,46]. In this period of time, the amount of small capillary pores decreases from 71.0% to 54.2% and, accordingly, large capillary pores reduce from 20.3% to 5.9%. The amount of capillary macropores with a diameter above 10 µm seems to be about constant for the duration of the experiment. The sealing of the microstructure caused by the pozzolanic reaction is strengthened up to day 360, when it slows down but does not seem to stop.

Figure 11 presents an overlay of the differential pore size distribution of FA mortars cured for varying lengths of time.

**Figure 12:**
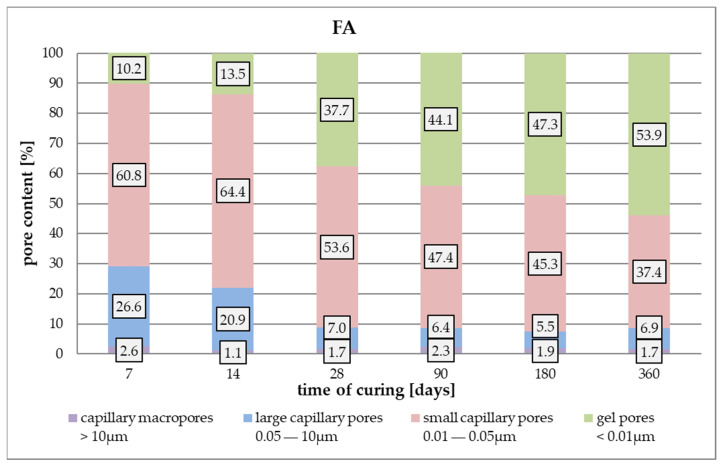
Data from the FA mortar tests.

Plots in Figure 11 present the changes in pore size distribution in FA mortars throughout the process of curing. The shape of the curves is similar to those registered for R-MUD mortars. Samples cured for up to 14 days have a relatively high number of large capillary pores ranging from 1 µm to 0.01 µm. Between days 14 and 28 of curing, pore size distribution rapidly changes and shows as the main process. Large capillary pores are transformed into small capillary pores and gel pores, with the highest number of pores having a diameter of about 0.07 µm. Further increasing curing time was successful in sealing the microstructure, decreasing the number of capillary pores and increasing the amount of gel pores. Figure 12 presents the relevant experimental results.

Figure 12 presents how the amount of each type of pores changes in total porosity in FA mortars throughout curing. The type of pore size transformation is similar to that observed in R-MUD but in FA samples, it seems to be slower. The process of sealing the microstructure of FA mortars also speeds up before day 14 of curing, when the amount of gel pores increases from 13.5 to 37.7% for days 14 and 28, respectively. During this time, the amount of small capillary pores decreases from 64.4% to 53.6% and the large capillary pores reduce from 20.9% to 7.0%, accordingly. The amount of capillary macropores with a diameter of above 10 µm seems to be about constant throughout the experiment.

Comparing the results of the MIP tests between the R-MUD and FA mortars, it was observed that the process of sealing the microstructure was similar for both types of mortar. In R-MUD, changes in the pore size distribution between day 14 and day 28 of curing were more rapid, which might be explained by the increasing speed of the pozzolanic reaction. The hydration of clinker constituents was also considered but, according to the literature [23,49,50], the speed of the hydration reaction of the clinker slows down during this period of time and almost stops at day 28. The process of sealing the microstructure explained by the pozzolanic reaction intensifies up to day 360 for both types of mortar but in FA, it ultimately slows down and almost stops entirely. In R-MUD mortars, however, it does not seem to stop after a year of curing. This might be caused by the higher amount of constituents in R-MUD that are likely to react during a pozzolanic reaction or by their increased effectiveness, as is the case with colloidal silicone, as well as the lower content of total silicon and aluminium oxides present in the R-MUD.

### 3.5. Scanning Electron Microscopy

The aim of SEM examinations was to analyse the microstructure of R-MUD and FA mortars cured for 360 days focusing on the sealing properties of the C-S-H phase and various types of transition zones between grout and aggregate, fly ash and R-MUD constituents.

#### 3.5.1. R-MUD Mortars

**Figure 13:**
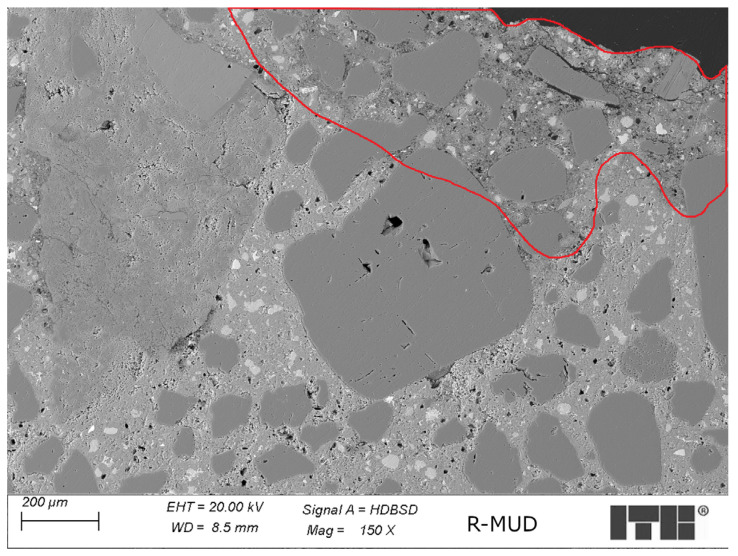
Example of the microstructure of R-MUD mortar cured for 360 days—marked areas of less sealed microstructure.

Figure 13 presents the microstructure of R-MUD mortar cured for 360 days.

The observed microstructure of the R-MUD mortar cured for 360 days was sealed and dense with the presence of macropores, which were not fully overgrown by the products of cement hydration. The pores were surrounded by the C-S-H phase, which was well sealed and well developed. This might explain why they were not fully detected in the MIP tests and not included into the pore size distribution [51]. Some areas of the cement matrix in the examined mortar are more sealed than others, as shown in Figure 13. This might be explained by the fact that the cement paste is not fully homogeneous, and its composition might differ across regions. This also influences the effectiveness of cement hydration and the pozzolanic reaction. The areas of better sealed cement matrix were larger than areas with the more porous C-S-H phase.

**Figure 14:**
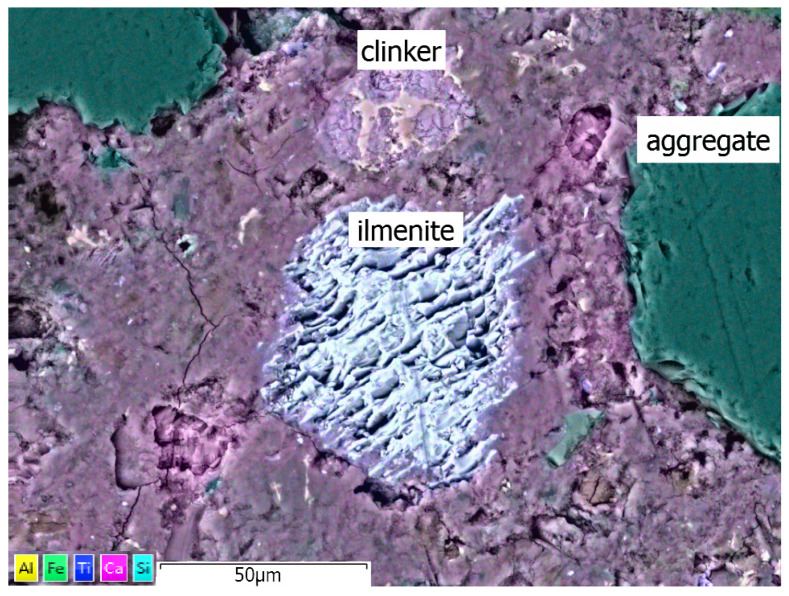
EDX map of R-MUD mortar microstructure.

Some relics of clinker are also present in the cement matrix, as shown in Figure 14. They were observed in areas of sealed structure with the C-S-H gel. The constituents of R-MUD, such as ilmenite grains, were well built into the cement matrix. The C-S-H phase close to the transition zone was enriched with titanium content. Also, the migration of calcium ions inside ilmenite grains was observed (purple halo inside ilmenite grains, as seen in Figure 14). The transition zone between the cement matrix and the aggregates was well developed and sealed in most of the observed areas. The C-S-H phase near the transition zone was enriched with silicon from the aggregate grains. No signs of any corrosion processes were observed.

#### 3.5.2. FA Mortars

**Figure 15:**
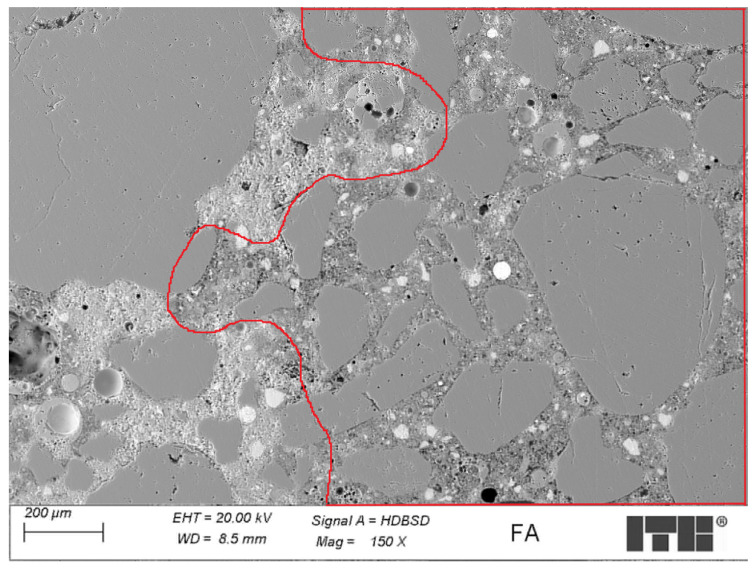
Example of the microstructure of FA mortar cured for 360 days—marked area of less sealed microstructures.

Figure 15 presents an example of the microstructure of FA mortar cured for 360 days.

The examination of the microstructure of FA mortars cured for 360 days has shown that it was well sealed and dense at a level similar to the R-MUD mortar. The cement matrix was sealed; however, areas that were more or less sealed were observed. Areas of the C-S-H phase with higher porosity seem to be more frequent than more sealed areas, which is also related to the MIP tests. The location of looser microstructures, found mostly under the grains of aggregates, suggests the uneven concentration of water in the cement matrix. The water–cement ratio was higher under the large aggregate grains than above them, which led to differences in porosity. In the cement matrix, macropores were also present and surrounded by the sealed structure of the C-S-H phase, which might not be fully detected by the MIP method. Also, the larger grains of fly ash were porous but their porosity was probably not included in the MIP tests due to their dense structure and the sealed structure of the surrounding cement matrix, which does not allow for the introduction of mercury during the MIP tests [51].

**Figure 16:**
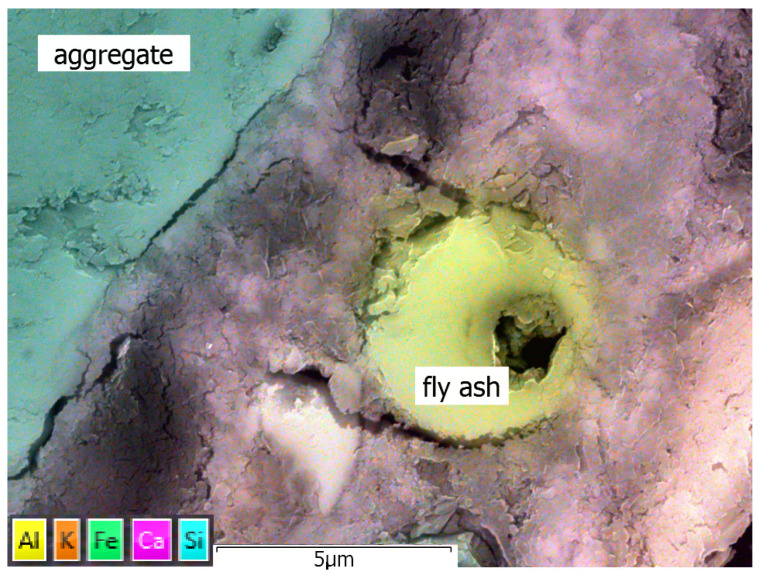
EDX map of FA mortar microstructure.

Figure 16 presents an example of an EDX map of FA mortar showing the fly ash grains surrounded by the sealed C-S-H phase. Visible cracks occurred during the SEM sample preparation related mostly to the drying process, which led to the partial dehydration of the C-S-H gel. The transition zone between fly ash grains and the cement matrix was sealed and well developed, and the grains were well bound into the cement matrix. The migration of aluminium (yellow halo outside fly ash grains) and calcium ions (purple halo inside fly ash grains) across the transition zone shows the pozzolanic activity of the fly ash’s constituents. Figure 15 includes part of an aggregate grain in contact with the cement matrix. The transition zone is well developed and mostly sealed. The cement matrix in the areas close to the transition zone has a higher concentration of silicon ions, shown as a blue halo at the right side of the observed part of the aggregate grain. No signs of any corrosion processes were observed.

Comparing the microstructures of R-MUD and FA mortars cured for 360 days, examined by SEM, led to the following observations. The microstructure of both mortars was sealed and very similar in general to one another. Both mortars had areas with more and less sealed microstructures of C-S-H gel; however, in the R-MUD mortar, sealed areas dominated while the opposite was true for FA mortar, where less sealed areas dominated. In both types of mortar, a number of macropores was observed, which might not be detectable by the MIP method due to the surrounding sealed C-S-H gel that does not allow for the introduction of mercury during the MIP tests. Transition zones between the C-S-H gel and several types of other constituents, e.g., aggregates, ilmenite and fly ash, were sealed, and for the most part, well developed and bound to the cement matrix, showing the migration of ions from the grains to the C-S-H gel and, on occasion, also in the opposite direction.

## 4. Conclusions

Analysis of the test results focused on the influence of pozzolanic reactivity of R-MUD on the observed microstructure of mortar compared to FA mortar. The following conclusions were reached:

- R-MUD has a lower content of silicon dioxide and aluminium oxide than fly ash; however, it seems to be more active than FA. This might be caused by the higher content of colloidal silicon dioxide which is not present in fly ash.
- R-MUD is a pozzolanic active material, which seals the microstructure of cement composites more effectively than fly ash.
- The pozzolanic reaction of R-MUD seems to subsist beyond 360 days, unlike fly ash where pozzolanic activity ceases after a year.
- Results of R^3^ bound water tests proves the pozzolanic activity of R-MUD although differences between results of tests received using other methods needs further investigations as it might be that not all methods are accurate for testing this type of material.

R-MUD thus promises to be a useful substitute of FA in cement composites. The pozzolanic activity of R-MUD was assessed by its impact on the microstructure of the cement matrix, in comparison to the undoubted pozzolanic activity of FA. It has shown that R-MUD is a pozzolanic reactive material and its properties in this area are similar to the FA; however, the mechanism seems to be slightly different. R-MUD contains less silicon dioxide than the FA, although it probably contains the colloidal form of silicon dioxide which reacts very effectively. The mechanisms of its higher pozzolanic activity and in particular, the presence of colloidal silicon, however, need to be further investigated.

## Figures and Tables

**Table 1:** Composition of R-MUD, FA, and cement [31,33].

Material	Constituent Content [%]												Relevant Surface [cm^2^/g]
	SiO_2_	TiO_2_	Fe_2_O_3_	MgO	Al_2_O_3_	CaO	Na_2_O	MnO	K_2_O	P_2_O_5_	SO_3_	Cl	
R-MUD	35.07	33.05	9.65	7.26	5.53	3.09	1.10	0.53	0.26	0.01	0.98	–	8390
Fly ash	51.51	1.09	8.51	2.53	25.71	3.82	1.37	0.10	2.73	0.31	0.48	0.02	4020
Cement CEM I 42.5R	20.06	–	3.38	0.89	4.13	64.41	0.24	–	0.56	–	2.97	0.07	4060

**Table 2:** Composition of tested mortars.

Constituent	Mortar R-MUD	Mortar FA
cement CEM I 42.5R [g]	401.4	401.4
R-MUD/FA [g]	48.6	48.6
sand [g]	1350	1350
water (water/binder) [g]	225 (0.5)	225 (0.5)

**Table 3:** Results of the R^3^ tests.

Sample	Bound Water [g/100 g Dried Paste]
R-MUD	6.12
FA	7.53

## Data Availability

The original contributions presented in the study are included in the article, further inquiries can be directed to the author.
